# Quantitative Investigation into the influence of intravenous fluids on human immune and cancer cell lines

**DOI:** 10.1038/s41598-020-61296-5

**Published:** 2020-07-16

**Authors:** Hande Karamahmutoglu, Alara Altay, Sumeyra Vural, Meltem Elitas

**Affiliations:** 1 Faculty of Engineering and Natural Sciences, Sabanci University, Istanbul, 34956 Turkey; 2 Sabanci University Nanotechnology and Application Center, Sabanci University, Istanbul, 34956 Turkey

**Keywords:** Breast cancer, Hepatocellular carcinoma

## Abstract

The effect of intravenous fluids (IVF) has been investigated clinically through the assessment of post-treatment reactions. However, the responses to IVF vary from patient-to-patient. It is important to understand the response of IVF treatment to be able to provide optimal IVF care. Herein, we investigated the impact of commonly used IVFs, Dextrose, NaCl and Ringer on different human cancer (HepG2 (liver hepatocellular carcinoma) and MCF7 (breast adenocarcinoma)) and immune cell lines (U937 (lymphoma) monocyte and macrophages). The effect of IVF exposure on single cells was characterized using hemocytometer, fluorescence microscopy and flow cytometry. Quantitative data on the viability and morphology of the cells were obtained. Our results emphasize that different IVFs demonstrate important differences in how they influence distinct cell lines. Particularly, we observed that the lactated ringer and dextrose solutions altered the viability and nuclear size of cancer and immune cells differently. Our findings present valuable information to the knowledge of cellular-level IVF effects for further investigations in IVF usage on diverse patient populations and support the importance and necessity of developing optimal diluents not only for drug stability but also for patient benefits.

## Introduction

The choice of IVF selection has been debated over the years. IVF therapy is one of the main treatment methods that have been used to cure water imbalance problems^1^. This technique involves the application of intravenous fluids (IVF). The first usage of IVF dates to the 1830s when Shaughnessy used intravenous salt solutions to treat cholera^2,3^. Since then, IVF, have been utilized in clinical practice on a large scale through intravenously injections to patients^4^. Over the years, discussions regarding the choice of IVF selection^5–8^ procedures of IVF that were accepted as standard for patient treatment have shifted to personalized fluid treatment depending on patients’ conditions^9,10^. Several types of IVF including normal saline (NS), dextrose and ringer have been commonly used^11,12^. In order to investigate IVF therapy, mostly to test diluents, which are optimal for drug compatibility and stability, experiments have been presented^13^.

First human trial was performed to assess the immunologic and anti-inflammatory impact of hypertonic resuscitation in trauma patients. The results showed that hypertonic saline plus dextran leaded to a more balanced inflammatory response to hemorrhagic shock^14^. To better understand the effects of IVF on patients, quantitative analysis might contribute notably to a better comprehension of cellular individuality and heterogeneity^15^. As vascular endothelial cells form the primary selective barrier to plasma macromolecules, support red blood cell movement, oppose transvascular fluid flux and leukocyte adhesion, majority of the quantitative studies focused on investigating response of blood cells and endothelial cells to IVF. Carden’s group reported the influence of IVF on sickle cell disease. They measured the stiffness of the red blood cells in 0.45% and 0.9% sodium chloride solutions at single-cell level. They concluded that sodium chloride solutions alter biomechanical properties of sickle red blood cells. Consequently, patients with sickle cell disease might have localized microvascular obstruction during the vasso-oclusive crisis^16^. Koustova and her co-workers quantified the production of reactive oxygen species from leukocytes when they were exposed to Lactated Ringer solution. They observed elevated level of reactive oxygen species and concluded that it influenced neutrophil function and leukocyte gene expression^17^. Jackson and Derleth examined the effect of brief contact with various infusion solutions on red blood cells from newborn infants. They determined that isotonic amino acid solution did not cause agglutination or haemolysis difference^11^. Likewise, Shields *et al*., quantified the adhesion molecules and laminin expression to reveal influence of hypertonic saline solution to the tumor cell-endothelial cell interaction^18^. Dr. Schmidt’s group employed mass spectrometry to measure plasma heparan sulfate, which is associated with volume of intravenous fluids, and determined that intravenous fluid resuscitation is associated with septic endothelial glycocalyx degradation^19^.

The study presented here aimed to provide valuable insights into the distinct cellular responses of monocytic leukemia and carcinoma cells induced by IVF. Epithelial cells function as barrier to pathogens, express a wide variety of immunomodulatory molecules and play a crucial role in the recognition and resolution of inflammatory responses. Our study designed to interrogate the acute response of U937 monocytes, U937 monocyte-derived macrophages^20^, HepG2 human liver hepatocellular carcinoma cells^21^, and MCF7 human breast adenocarcinoma cells^22^ when they were exposed to various IVF for 15 minutes. We used immune system related cell lines, U937 monocyte and U937-differentiated macrophage due to their heterogeneity and abundance in blood^23^. Although clinical studies demonstrated that IVF modulates macrophage response and hypertonic saline resuscitation decreases alveolar macrophage activation, cell viability and morphologic changes have not been reported^24^. As cancer cell lines, we chose MCF7, which might be one the most metastatic cancer line^25^ and HepG2, whose dominant risk factors reflect the worldwide heterogeneous incidences such hepatitis (B, C) viruses, alcoholism, aflatoxin, obesity, diabetes, cirrhosis^26^. In order to quantify the influence of IVF on these human cell lines, we performed single-cell resolution viability assays using hemocytometry, fluorescent microscopy, and flow cytometry. It is important to emphasize that limited studies have been conducted to quantitatively investigate cellular, metabolic, and systematic consequences of IVF on human monocytes and epithelial tissue cells. Miltra’s group employed RNA sequencing to explore global transcriptional responses induced by hypertonic saline solution^27^. Their results suggested that hypertonic saline attenuates the cytokine-induced pro-inflammatory signature in primary human lung epithelia^27^. Likewise, Dr. Banerjee and co-workers measured inflammatory cytokines and chemokines in response to TNF-α and IL-1β on alveolar pneumocyte line (A549) and concluded that hypertonic saline or hyperosmolar media disrupt cytokine signals at distinct intracellular steps^28^. Similarly, this group previously reported that hypertonic saline inhibited TNF-α induced NF-κB activation in the pulmonary epithelium using immunofluorescent microscopy, flow cytometry, cell viability and western blot analysis^29^.

However, still most of the recent discussions focus on investigating large clinical trials to better define optimal fluid therapy in acute and critical care medicine^30–34^.

## Results

We observed the acute response of U937 monocytes, U937-derived macrophages, MCF7 and HepG2 cancer cells when we exposed them to IVF for 15 minutes. We quantified the number of live and dead cells using hemocytometry, inverted fluorescent microscopy and flow cytometry data as described in the materials and methods section. Our results displayed the cellular and nuclear area adaptations and viability counts of the cells when the cells were incubated in IVF, PBS and each cell’s respective medium.

**Figure 1:**
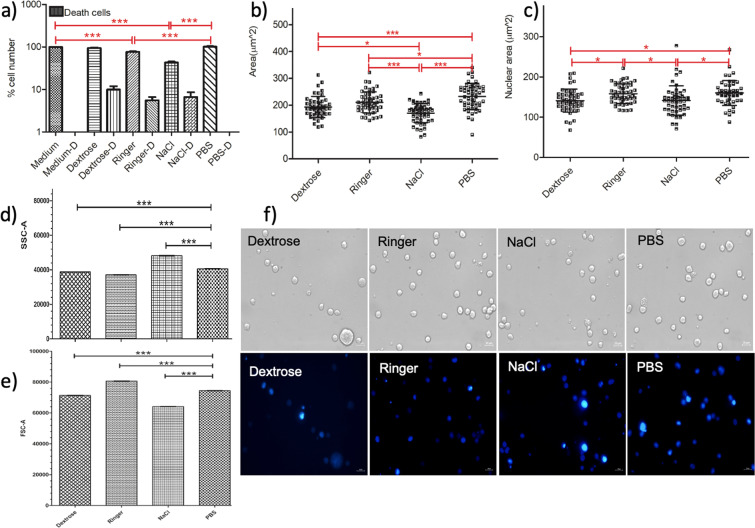
Viability and cellular area of the U937 monocyte cells decrease in IVF. (**a**) Cell viability graph of U937 monocytes showing the percentage cell number after incubation in IVF, medium and PBS for 15 minutes. (**b**) Surface area measurements of the U937 monocytes presented upon IVF treatment, medium and PBS incubations for 15 minutes. (**c**) Analysis of the nucleus size with mean and standard deviations after 15 minutes incubation in Dextrose, NaCl, Ringer and PBS. One-way ANOVA Tukey’s Multiple Comparison Test is applied for (**a–c**), p < 0.05 is significant. (**d**) Side scatter and (**d**) forward scatter comparisons using Student’s t-test, based on flow cytometry data presented in the Supplementary Material (Fig. S1). (**f**) The phase and fluorescence microscopy images of the DAPI stained U937 monocytes are obtained by the inverted fluorescent microscope after 15-minute incubation in PBS, Dextrose, Ringer and NaCl with exposure times: 12,5 ms and 300 ms (DAPI). The scale bar shows 20 µm. Data are representative of three independent experiments and the values are expressed in mean ± s.d.

First, we quantified the number of live and dead cells for the U937 monocytes, U937-derived macrophages, MCF7, and HepG2 cancer cell lines after 15-minute incubation with IVF. Figure 1 shows the response of U937 monocytes to IVF. Figure 1a represents the percentage viability of U937 cells in IVF in comparison to in PBS based on the hemocytometry data. Figure 1b illustrates the surface area changes, Fig. 1c demonstrates the nucleus size alterations of the U937 cells using the microscopy images shown in Fig. 1f. Figure 1d presents the side scatter, Fig. 1e shows the forward scatter comparisons using Student’s t-test, based on flow cytometry data presented in the Supplementary Material (Fig. S1).

**Figure 2:**
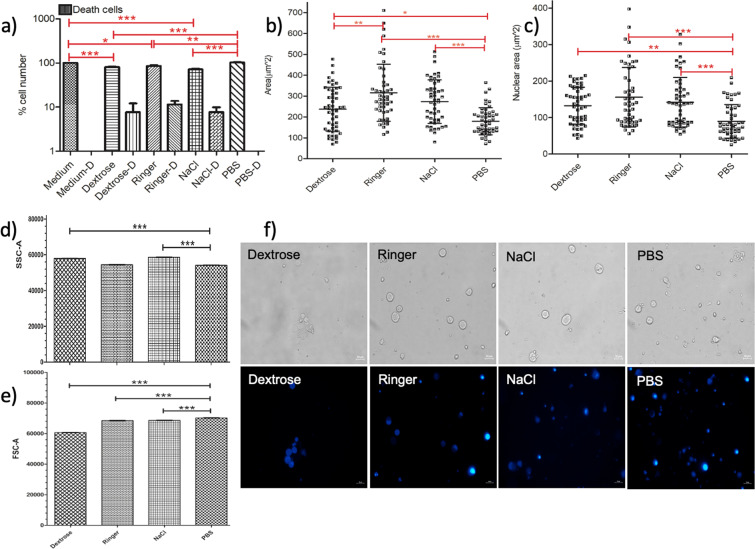
Viability of the U937-differentiated macrophages decrease, and their cellular area increased in the IVF solutions. (**a**) Cell viability graph of macrophages showing the percentage cell number after incubation in IVF, medium and PBS for 15 minutes. (**b**) Surface area, (**c**) nucleus size measurements of the macrophages presented upon IVF treatment, medium and PBS incubations for 15 minutes. One-way ANOVA Tukey’s Multiple Comparison Test is applied for (**a–c**), p < 0.05 is significant. (**d**) Side scatter and (**e**) forward scatter comparisons using Student’s t-test, based on flow cytometry data presented in Supplementary Material (Fig. S2). (**f**) The phase and fluorescence microscopy images of the DAPI stained U937 macrophages are obtained by the inverted fluorescent microscope after 15-minute incubation in PBS, Dextrose, Ringer and NaCl with exposure times: 12,5 ms and 300 ms (DAPI). The scale bar shows 20 µm. Data are representative of three independent experiments and the values are expressed in mean ± s.d.

Figure 2 illustrates the acute response of U937-differentiated macrophage cells upon IVF treatment. Figure 2a shows the statistical analysis for the percentage cell number changes of U937-differentiated macrophage cells using a hemocytometer. Figure 2b demonstrates the distribution of the cellular areas and Fig. 2c demonstrates the analysis of the nucleus size using the microscopy images presented in Fig. 2f. Figure 2d presents the side scatter, Fig. 2e displays the forward scatter comparisons using Student’s t-test, based on flow cytometry data presented in the Supplementary Material (Fig. S2).

**Figure 3:**
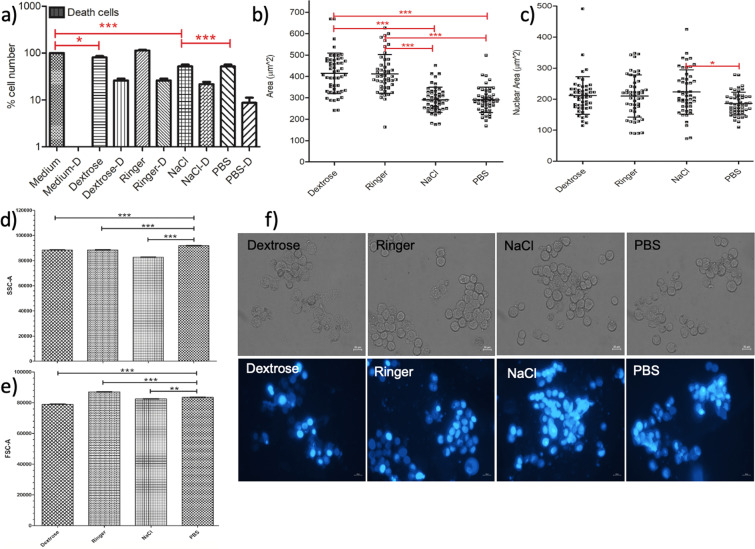
NaCl significantly decreases the viability of the HepG2 cells. (**a**) Cell viability graph of HepG2 cell line showing the percentage cell number after 15-minute incubation in IVF, medium and PBS. (**b**) Surface area, (**c**) nucleus size measurements of the HepG2 cells presented upon IVF treatment, medium and PBS incubations for 15 minutes. One-way ANOVA Tukey’s Multiple Comparison Test is applied for (**a–c**), p < 0.05 is significant. (**d**) Side scatter and (**e**) forward scatter comparisons using Student’s t-test, based on flow cytometry data presented in the Supplementary Material (Fig. S3). (**f**) The phase and fluorescence microscopy images of the DAPI stained HepG2 cells are obtained by the inverted fluorescent microscope after 15-minute incubation in PBS, Dextrose, Ringer and NaCl with exposure times: 12,5 ms and 300 ms (DAPI). The scale bar shows 20 µm. Data are representative of three independent experiments and the values are expressed in mean ± s.d.

We quantified the behavior of the HepG2 cells after 15-minutes IVF treatment, Fig. 3. The plots for the analysis of the flow cytometry data can be found in the Supplementary Material, (Fig. S3).

**Figure 4:**
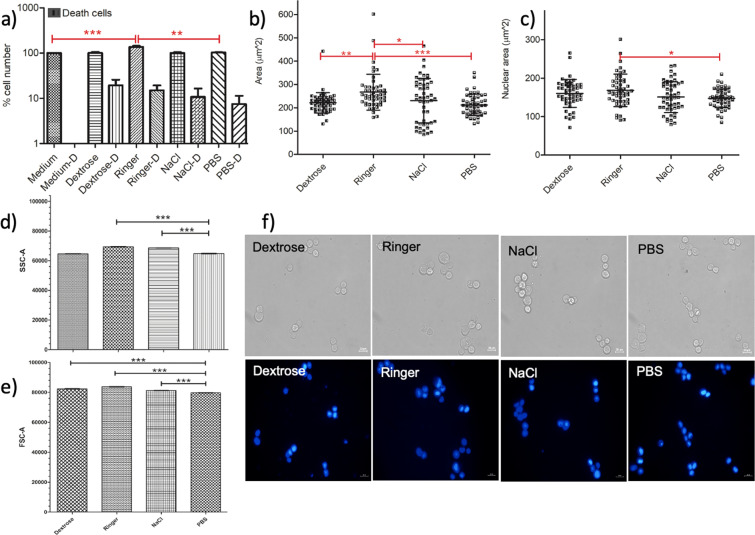
Ringer increases the viability, cellular area and nuclear area of the MCF7 cells. (**a**) Cell viability graph of MCF7 cell line showing the percentage cell number after incubation in IVF, medium and PBS for 15 minutes. (**b**) Surface area measurements of the MCF7 cells presented upon IVF treatment, medium and PBS incubations for 15 minutes. (**c**) Analysis of the nucleus size with mean and standard deviations after 15 minutes incubation in Dextrose, NaCl, Ringer and PBS. One-way ANOVA Tukey’s Multiple Comparison Test is applied for (**a–c**), p < 0.05 is significant. (**d**) Side scatter and (**e**) forward scatter comparisons using Student’s t-test, based on flow cytometry data presented in Supplementary Material (Fig. S4). (**f**) The phase and fluorescence microscopy images of the DAPI stained MCF7 cells are obtained by the inverted fluorescent microscope after 15-minute incubation in PBS, Dextrose, Ringer and NaCl with exposure times: 12,5 ms and 300 ms (DAPI). The scale bar shows 20 µm. Data are representative of three independent experiments and the values are expressed in mean ± s.d.

MCF7 cell viability and morphology assay results were demonstrated in Fig. 4. The flow cytometry data can be found in the Supplementary Material.

Based on the results of the One-way ANOVA Tukey’s Multiple Comparison Test, Tables 1–3 list the behavior of U937 monocyte, U937-differentiated macrophage, HepG2, and MCF7 cells. The statistical analysis of the forward and side scatter data obtained using flow cytometry data that can be found in the Supplementary Material.

**Table 1:** Ringer increases viability of MCF7 cancer cells.

Viability	Dextrose	Ringer	NaCl	Medium
U937 Monocyte	ns	***	***	ns
U937-differentiated Macrophage	***	**	***	ns
HepG2	ns	ns	***	ns
MCF7	ns	**	ns	ns

One-way ANOVA Tukey’s Multiple Comparison Test for the viability of U937 monocytes, macrophages, HepG2, and MCF7 cells in IVF relative to PBS. p < 0.05 is significant.

**Table 2:** Cellular and nuclear areas of the cells affected by IVF.

Area measurements	Dextrose		Ringer		NaCl	
	Surface	Nucleus	Surface	Nucleus	Surface	Nucleus
U937 Monocyte	***	*	*	ns	***	*
U937-differentiated Macrophage	*	**	***	***	***	***
HepG2	***	ns	***	ns	ns	*
MCF7	ns	ns	***	*	ns	ns

One-way ANOVA Tukey’s Multiple Comparison Test for the cellular area and nuclear area measurements of the U937 monocytes, macrophages, HepG2, and MCF7 cells in IVF relative to PBS. p < 0.05 is significant.

**Table 3:** Comparison of *in vitro* studies for IVF.

IVF	Tested cell line	Result	Method (in vitro)	Question	Reference
Hypertonic saline (HTS)	Human colon cancer cells (LS174T), Human umbilical vein endothelial cells (HUVECS)	HTS reduces adhesion molecule and laminin expression. HTS did not alter cell viability.	Flow cytometry, fluorescence microscopy	How does hypertonic saline contribute metastasis?	^18^
Hypertonic saline (HTS)	Primary human small airway epithelial cells (SAECS)	Hypertonic saline attenuates the cytokine-induced pro-inflammatory signature in primary human lung epithelia.	RNAseq, enzyme-linked immunosorbent assay, quantitative real-time polymerase chain reaction, luciferase assay, western blot, migration assay	How does HTS affect the whole transcriptome?	^27^
Hypertonic saline (HTS), Hyperosmolar media (HOsm)	Human pulmonary epithelial cells (A549)	Anti-inflammatory mechanisms of HTS/HOsm disrupt cytokine signals at distinct intracellular steps.	Quantitative real-time polymerase chain reaction, western blot, enzyme-linked immunosorbent assay, multiplex bead-bead assay, fluorescence microscopy	What is the role and mechanism of HTS inhibition in the presence of TNFα and IL-1β stimulation?	^28^
Hypertonic saline (HTS)	Human pulmonary epithelial cells (A549)	HTS inhibits TNF-α-induced NF-κB activation in the pulmonary epithelium.	Fluorescence microscopy, Western blot, flow cytometry, MTT assay	Does HTS inhibit TNF-α-induced NF-κB activation in the pulmonary epithelium?	^29^
Plasma-activated lactated Ringer (PAL)	Human pancreatic cancer cells (Capan-2, BxPC-2, AsPC-1/CMV-Luc, MIA PaCa-2)	PAL induces apoptosis in pancreatic cancer cells.	Microscopy, Absorbance. In vivo: Mouse model (BALB/c Slc-nu/nu mice)	Is PAL convenient to be used as a therapeutic for peritoneal metastasis?	^40^
Hypertonic saline (HTS)	Brain endothelial cells (bEnd.3)	40 mmol/L NaCl HTS enhances cell viability and attenuates cell apoptosis.	Flow cytometry, RNA-seq, Quantitative real-time polymerase chain reaction, western blot, enzyme-linked immunosorbent assay	How do the levels of IL-1β and EGFR is correlated to the performance of HTS and apoptosis?	^41^
Hypertonic saline (HTS)	HeLa cells	Increasing NaCl concentration more than 130 mM decreases growth rate, increases protein levels, and cell volume.	Biochemical analysis, hemocytometry, microscopy	Ionic and osmotic effects of HTS in the metabolism of HeLa cells	^50^
Hypertonic sucrose (HTSu)	Human leukemia cells(U937)	U937 cells developed apoptosis thanks to regulatory volume increase in HTSu.	Percoll gradient, ion concentration measurement, microscopy	Are U937 cells capable of being a model organism to study cellular volume regulation?	^51^
Hypertonic D-glucose (HTD-g)	Human breast cancer cells (MCF7)	HTD-g reduces the viability, increases the apoptosis and DNA damage of the MCF7 cells	MTT assay, Comet assay, flow cytometry	Does HTD-g induce cytotoxic, genotoxic, and apoptotic effects in tumor cells?	^52^

Flow cytometry experiments were performed to analyze a larger number of cells to verify single-cell analysis obtained using hemocytometer and microscopy images. We quantified the viability of the cells via Propidium Iodide (PI) staining. We analyzed morphology of the cells using forward scatter and side scatter data (See Supplementary Material).

## Discussion

IVF medication has been performed for millions of patients each year. Recently, new cell-based treatment strategies have been developed in which IVF plays a crucial role. Therefore, investigations of the effect of IVF have begun to emerge. The concerns of IVF on patient outcomes has been investigated in relation to critical care practice^35–39^. Most IVF investigations have focused on revealing their influence on medication stability, compatibility and administration^18^. To date, there have been few quantitative *in vitro* studies about the influence of IVF on cellular level, which are mostly reported for blood cells and endothelial cells^18,40,41^.

Here, we emphasized the acute effect of IVF currently used in the clinic, Dextrose, NaCl and Ringer, on two representative human immune cell lines (U937, monocytic leukemic cells and U937-derived macrophages) and two epithelial cell lines (HepG2, liver hepatocellular carcinoma epithelial cells and MCF7, breast adenocarcinoma epithelial cells). We characterized cell viability, cellular area and nuclear morphologic changes using multiple techniques to yield quantitative results that might highlight some potential cautions in using these IVF diluents in clinical disease management. Our results present *in vitro* evaluation for acute reactions of white blood cells and carcinoma cells when they were exposed to IVF for 15 minutes. Cell viability changes for 60-minutes IVF exposure were also observed, which is beyond the scope of this work (Table S3, Supplementary Material). Drastic morphological alterations and cell viability changes were noticed when the cells treated with IVF for 15 minutes. Our single-cell analysis results, reflecting a very short-term acute response, showed that the viability of the immune cells decreased by more than that of cancer cells when they were exposed to IVF for 15 minutes (Table 1).

Among IVF treatments, dextrose solution is one of the most commonly used diluents in clinics. However, recent studies have reported a growing uncertainty over the safety of dextrose solution^37^. Since dextrose is a form of glucose, which might alter cellular ability to sustain ion and water balance, intracellular homeostasis. Thus, dextrose solution might rupture the plasma membrane, causing cytoplasm leakage into the extracellular environment^42^. Our results showed that dextrose solution decreased the viability of the U937-differentiated macrophage cells (Figs. 1–4, Table 1). Cellular and nuclear area of macrophage cells increased in dextrose solution, but it did not significantly influence MCF7 cells (Figs. 1–4, Tables 1 and 2, see Supplementary Material).

The NaCl diluent is the other primary diluent in clinics; however, it has been reported that it might cause hyperchloremia, metabolic acidosis, acute kidney injury and renal vasoconstriction^38^. Since NaCl diluent makes the medium hypertonic^36^, it supports viability assays resulting in a decrease of cell counts, excluding MCF7 cells. Our results demonstrated that NaCl significantly decreased viability of U937 monocytes, macrophages and HepG2 cells, Figs. 1–4, Tables 1 and 2. Moreover, neither the cellular nor the nuclear areas of the MCF7 cells were altered in NaCl diluent, whereas others were affected, Figs. 1–4, Tables 1 and 2, and see Supplementary Material.

According to intensive care units, despite NaCl and dextrose being the main crystalloid fluid for revival, the utilization of lactated Ringer has been increased recently^7,31,32^. Dr. Brown and co-worker’s discussion about the outcomes of the medication diluent recommended balanced crystalloids such as lactated Ringer solution due to their chemical composition that is safe for a patient’s acid-base status and organ function^37,38^. In our study, the viability of immune related cells, U937 monocytes, and U937-differentiated macrophages decreased while the viability of breast cancer cells increased in Ringer solution (Figs. 1–4, Tables 1 and 2). Cellular and nuclear areas of the cells mainly became larger in the Ringer solution, except for U937 monocyte cells, their cellular size decreased, and nuclear size was not significantly altered (Figs. 1–4, Tables 1 and 2, see Supplementary Material).

In this study, we observed that IVF reduced the viability of immune cells more than cancer cells, Figs. 1–4. IVF change cellular or nuclear areas of the cells (Tables 1 and 2, see Supplementary Material). Kadota *et al*. reported that the nuclear diameter of the cells is one of the independent prognostic factors for worse outcomes when they performed comprehensive pathological analysis in lung squamous cell carcinoma^43^. Cell morphology and surface are crucial biomechanical properties for leading cellular behaviors like adhesion, spread and migration^18,44^. Based on our results, IVF might also lead to variation of nuclear diameter when patients are administered medication via IVF. Particularly for the lactated Ringer diluent, a previous study by Bonuccelli *et al*., in which MCF7 was tested in a co-culture environment, showed a risk of advanced metastasis in cancer patients^45^. Therefore, the utility of IVF should be explored to eliminate the growing uncertainty over its safety and benefits.

Distinctly, in this study, along with investigating a very short-term acute IVF influence on cell viability, we examined the surface areas of cells and their nuclei performing image analysis on microscopy images^46^. However, the number of cells for these image analyses was relatively low; therefore, we performed flow cytometry analysis (see Supplementary Material). Nonetheless, one of the most important limitations of our study is that it exhibits *in vitro* behavior with IVF in cell lines that do not recapitulate exactly the interactions in the human body when the medication is delivered via IVF.

Our results lay groundwork for further studies. As a future work, these studies might be conducted using three-dimensional (3-D) organs-on-a-chip which might provide a better understanding of biokinetics, signaling between cells and clinical insights^47^. Currently, organotypic culture studies are also in their infancy and there is an urgent need for their improvement to achieve *in vivo* likeness with a better practical usage in clinical studies^48^.

By reflecting a very short-term acute influence of IVF on U937 monocytes, U937-differentiated macrophages, HepG2, and MCF7 cancer cells, it was discovered that the viability of the monocytes and macrophage has significantly decreased compared to those of breast and liver cancer cells. Moreover, IVF introduced variations in the nuclear and cellular area of the cells. Our findings should be interpreted with caution since they do not present the influence of IVF on cells in the human body. However, our study supports the urgent need for more accurate interrogation of IVF at high resolution and more physiologically relevant human body mimicking models.

## Methods

### Chemicals and methods

Cell culture media and supplements including Phorbol 12-myristate 13-acetate (PMA), dimethylsulfoxide (DMSO), Dulbecco’s phosphate buffered saline (PBS), Dulbecco’s modified Eagle’s medium (DMEM), Roswell Park Memorial Institute (RPMI 1640) and Trypsin (3X) were purchased from Pan Biotech (Germany). Fetal bovine serum (FBS) and penicillin/streptomycin were purchased from Sigma Aldrich (St. Louis, MO). The 75 cm^2^ cell culturing flasks were purchased from TTP (Switzerland). Sterile IVF were taken from Polifarma (Turkey); polifleks 9% isotonic NaCl solution (308 mOsm/L) in 250 mL package, 5% Dextrose solution (277.47 mOsm/L), and Lactated Ringer’s solution (275.52 mOsm/L) in 500 mL packages^49^. Stains of Hoechst (10 mg/L in water) and Propidium Iodide (PI, 1 mg/L in water) were received from Life Technologies and Sigma Aldrich, respectively. Authors must ensure that their Methods section includes adequate experimental and characterization data necessary for others in the field to reproduce their work.

### Cell culture

The immortalized human histiocytic lymphoma monocyte cell line, U937 (ATCC® CRL1593.2™), human liver hepatocellular carcinoma epithelial cell line, HepG2 (ATCC® HB-8065™), and immortalized human breast adenocarcinoma epithelial cell line, MCF7 (ATCC® HTB-22™) were purchased from the ATCC (LGG Standards, Middlesex, UK). The human histiocytic lymphoma macrophages were differentiated from the U937 monocytes through incubation of 3 × 105 cells/mL in 5 mL RPMI 1640 with 10% FBS and 5 μL working solution including 10% PMA obtained from 10 ng/mL PMA/DMSO stock solution for 5 days. All cells were kept in 75 cm^2^ flasks in the incubator (NUVE, Turkey) maintaining a humidified atmosphere at 37 °C and 5% CO_2_. The U937-monocyte media was renewed every 3 to 4 days depending on cell density. The U937 monocyte-derived macrophage media was replaced every day until their usage. Both HepG2 and MCF7 cells were passaged every 3 to 4 days through a standardized trypsinization method using 0.25% (v/v) trypsin-EDTA in PBS depending on their confluency (85%) according to ATCC protocols.

### Cell viability assay using hemocytometer

We centrifuged and collected the U937 monocytes and U937-derived macrophages at 3000 rpm for 5 minutes, the MCF7 and HepG2 cell lines at 1800 rpm for 10 minutes. Next, using a hemocytometer (Marienfeld, Germany), we enumerated the viable and nonviable cells based on Trypan blue staining. We arranged the cell count 1.5 × 10^5^ cells/mL using their growth medium. Then we collected the cells using the centrifuge conditions stated above and resuspended the cells into 1 mL IVF; Dextrose, NaCl, Ringer and PBS, respectively. Subsequently, the tubes with different IVF were incubated for 15 minutes in the incubator. Upon incubation, viable and nonviable cells were enumerated using the hemocytometer. The experiments were conducted, as three independent experiments for each cell line. The maximum decrease in the viability of the cells was at the first 15 – minute time interval during the one-hour of IVF exposure. Therefore, our results present a very short-term acute response of the cells when the cells were incubated in IVF fluids for 15 minutes.

### Staining

Hoechst (0.128 mM) and PI (0.0111 mM) solutions were used for end-point staining. Cells were stained for 20 minutes in the incubator using 1 µL of dyes from the working solutions those explained in the chemicals and reagents above.

### Fluorescent microscopy imaging and single-cell analysis

To quantify the influence of IVF at single-cell resolution, the cells were prepared as explained above. 40 µL of cell suspension from each cell solution was sandwiched between two glass slides. The cells were observed on the inverted fluorescent microscope. Images of the cells were captured with a 40× objective using a Carl Zeiss, Axio Observer Z1 motorized stage equipped with the AxioCam Mrc5 camera. Both phase and fluorescent channels were used to obtain the images at 12,5 ms and 300 ms exposure times, respectively. We used both DsRed fluorescent and DAPI channels to visualize cytoplasm and nuclei of the cells. We counted the cell number and measured the area of the nuclei and cells using ImageJ on the acquired phase and fluorescent images.

### Flow cytometry analysis

We adjusted the number of cells to 2.5 × 105 cells/mL. We exposed the cells to the IVF for 15 minutes. We treated one of the samples with PBS and stained with PI, while the other samples were exposed to each IVF (Dextrose, NaCl, Ringer) and stained with PI. We performed the flow cytometry experiments using the BD LSRFortessa FACS analyzer (BD Biosciences, Franklin Lakes, NJ, USA). The intact cells were accomplished based on their forward scatter (cell size) versus side scatter (cell granularity) profiles using blue (488 nm) excitation laser. We analyzed the data using the FlowJo v10 software (TreeStar, Inc., OR, USA). We performed double cell discrimination by plotting the height against the area for forward scatter. While double cells have roughly the same height with single cells, they have double the area values of single cells. Thus, we identified and excluded double cells based on disproportions between height and area. Then, we distinguished live cells from debris and dead cells by gating on the area values of cells for forwards scatter versus side scatter plots. We excluded events found at the bottom left corner of the density plots in gating to separate debris and dead cells with lower level of forward scatter.

### Statistical analysis

The obtained image data was analyzed using GraphPad Prism software. We performed One-way ANOVA with Tukey’s Multiple Comparison test to compare viability, cellular and nuclear area variations of the cells when the cells were exposed to IVF for 15 minutes. The viability data was obtained from the hemocytometer, viable cells were label-free and nonviable cells were stained with Trypan blue. The area measurements of the cells were performed in ImageJ as explained above. Figures showed the data as mean ± standard deviation (SD) of three independent experiments. Statistical differences were represented with (*) for p < 0.05. The q values were the relevant critical values of the studentized range statistic.

## Supplementary information

Supplementary Information.
