# Supplementary material for: Quantitative Investigation into the influence of intravenous fluids on human immune and cancer cell lines
Source: Sci Rep. 2020 Jul 16;10:11792. doi: 10.1038/s41598-020-61296-5 (PMC7366617; doi:10.1038/s41598-020-61296-5)
Supplement: Supplementary file 1 — Supplementary Information. [file 41598_2020_61296_MOESM1_ESM.pdf]

Quantitative Investigation into the influence of intravenous fluids on human immune and cancer cell lines

Hande Karamahmutoglu[1,2], Alara Altay[1,2], Sumeyra Vural[1,2], Didem Ozkazanc[1,2], and Meltem Elitas[1,2,*]

[1]Faculty of Engineering and Natural Sciences, Sabanci University, Istanbul, 34956, Turkey
[2]Sabanci University Nanotechnology and Application Center, Sabanci University, Istanbul, 34956, Turkey

khande@sabanciuniv.edu
alaraaltay@sabanciuniv.edu
sumeyra@sabanciuniv.edu
didemozkazanc@sabanciuniv.edu
[*]melitas@sabanciuniv.edu

Supporting information

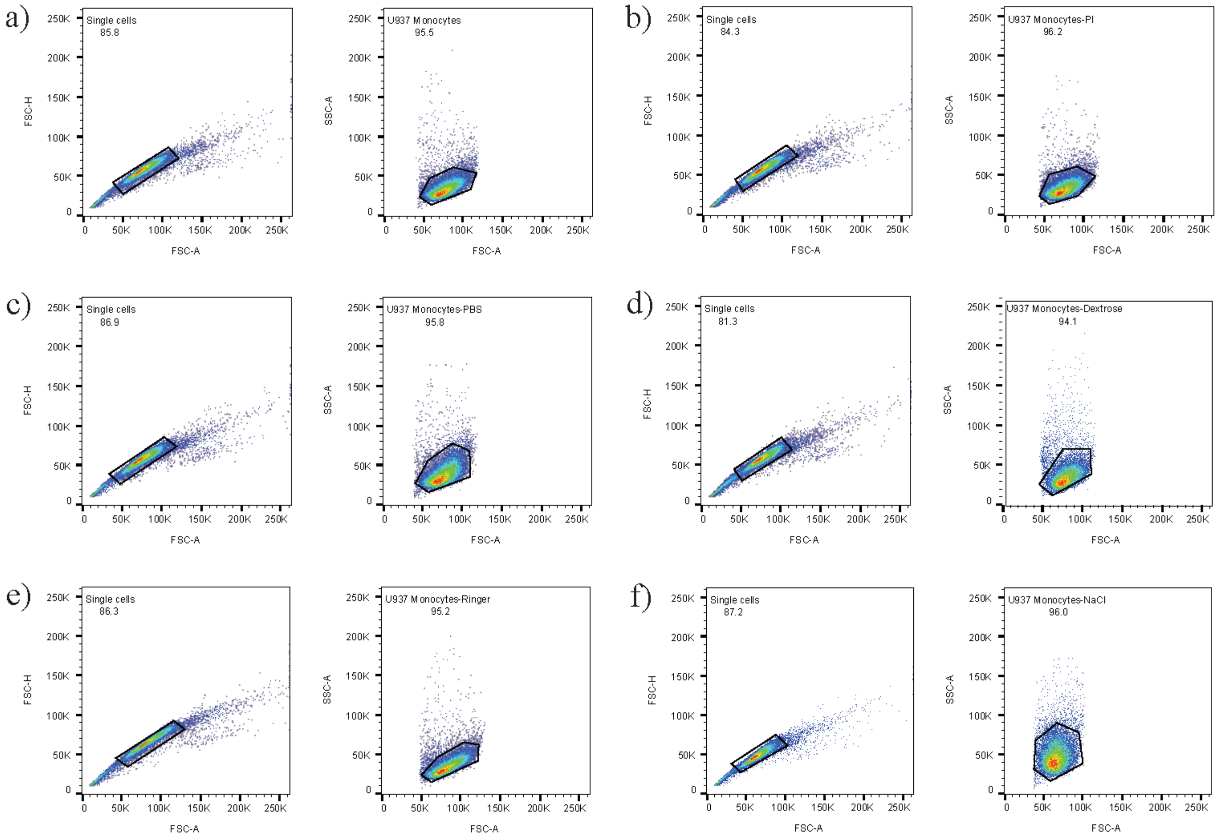

S1 Figure Forward scatter (FCS) vs. side scatter (SSC) data are depicted in the dot display mode, and the core population of the U937 cells is surrounded by a gate for confirming cell morphology changes in IV fluids. (a) The U937 monocytes without staining with PI and incubation in IV fluids. (b) U937 monocytes with PI staining and without incubation in IV fluids. U937 monocytes with PI staining and 15 minutes incubation in PBS (c), in Dextrose (d), in Ringer (e), in NaCl (f).

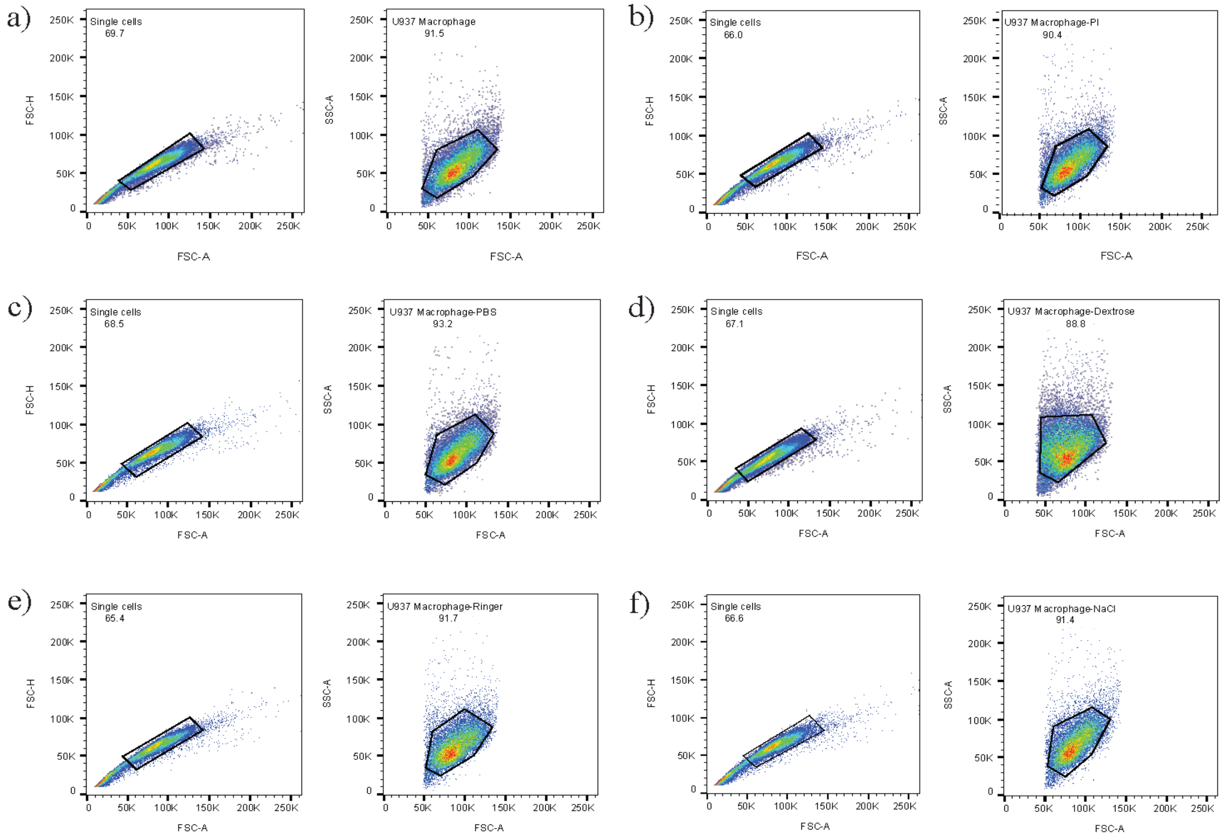

S2 Figure Forward scatter (FCS) vs. side scatter (SSC) data are depicted in the dot display mode, and the core population of the U937-differentiated macrophages is surrounded by a gate for confirming cell morphology changes in IVF. (a) The U937-differentiated macrophages without staining with PI and incubation in IV fluids. (b) U937 monocytes with PI staining and without incubation in IVF. Macrophages with PI staining and 15 minutes incubation in PBS (c), in Dextrose (d), in Ringer (e), in NaCl (f).

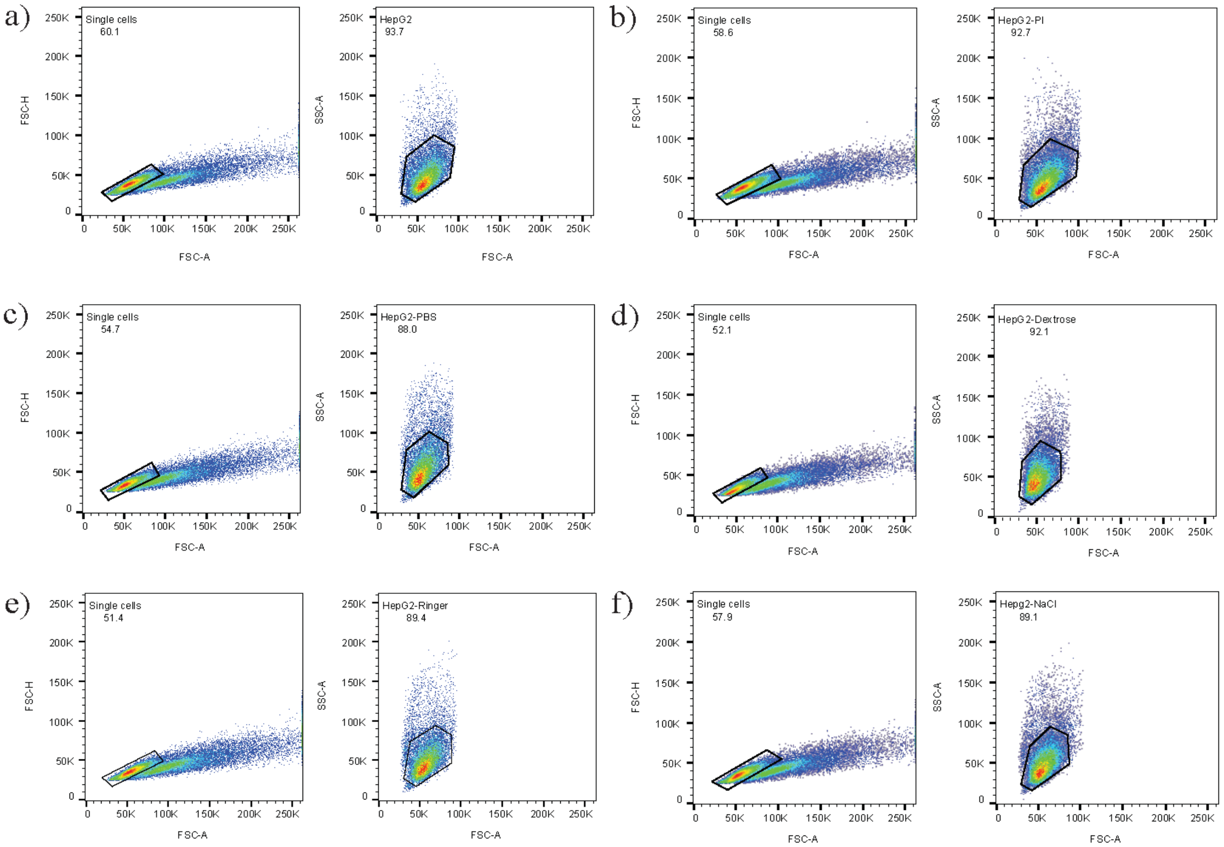

S3 Figure Forward scatter (FCS) vs. side scatter (SSC) data are depicted in the dot display mode, and the core population of the HepG2 cells is surrounded by a gate for confirming cell morphology changes in IV fluids. (a) The HepG2 cells without staining with PI and incubation in IV fluids. (b) The HepG2 cells with PI staining and without incubation in IV fluids. The HepG2 with PI staining and 15 minutes incubation in PBS (c), in Dextrose (d), in Ringer (e), in NaCl (f).

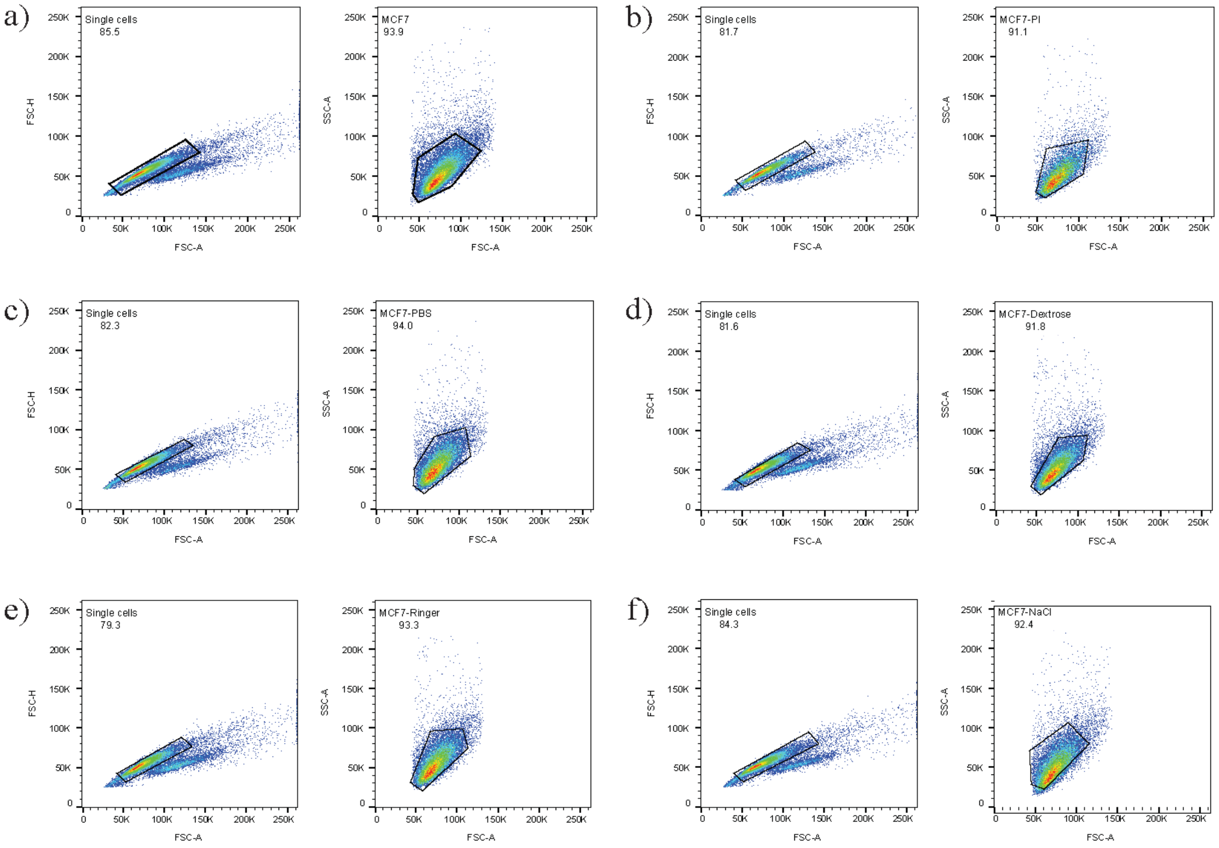

S4 Figure Forward scatter (FCS) vs. side scatter (SSC) data are depicted in the dot display mode, and the core population of the MCF7 cells is surrounded by a gate for confirming cell morphology changes in IV fluids. (a) The MCF7 cells without staining with PI and incubation in IV fluids. (b) The MCF7 cells with PI staining and without incubation in IV fluids. The MCF7 with PI staining and 15 minutes incubation in PBS (c), in Dextrose (d), in Ringer (e), in NaCl (f).

Table S1 Student's t-test results based on forward scatter comparison using flow cytometry data

**Cell Lines**	**Dextrose**	**Ringer**	**NaCl**
Monocyte	*** ($p< 0.0001$)	*** ($p< 0.0001$)	*** ($p< 0.0001$)
Macrophage	*** ($p< 0.0001$)	*** ($p< 0.0001$)	*** ($p< 0.0001$)
HepG2	*** ($p< 0.0001$)	*** ($p< 0.0001$)	** ($p=0.0013$)
MCF7	*** ($p< 0.0001$)	*** ($p< 0.0001$)	*** ($p< 0.0001$)

Table S2 Student's t-test results based on side scatter comparison using flow cytometry data

**Cell Lines**	**Dextrose**	**Ringer**	**NaCl**
**Monocyte**	*** ($p< 0.0001$)	*** ($p< 0.0001$)	*** ($p< 0.0001$)
**Macrophage**	*** ($p< 0.0001$)	ns ($p=0.2188$)	*** ($p< 0.0001$)
**HepG2**	*** ($p< 0.0001$)	*** ($p< 0.0001$)	*** ($p< 0.0001$)
**MCF7**	ns ($p<0.6259$)	*** ($p< 0.0001$)	*** ($p< 0.0001$)

Table S3 60 minutes exposure time for the cells in the IVFs

Cell Lines	Time (min)	Dextrose	Ringer	NaCl
**Monocyte (cells/ml)**	0	3.8×10^6	3.8×10^6	3.8×10^6
	60	10^6	7.5×10^5	7×10^5
**Macrophage (cells/ml)**	0	1.135×10^6	1.84×10^6	1.135×10^6
	60	2.4×10^5	2.2×10^5	2.5×10^5
**HepG2 (cells/ml)**	0	2.5×10^5	2.5×10^5	2.5×10^5
	60	2.5×10^5	3×10^5	6.5×10^5
**MCF7 (cells/ml)**	0	1.14×10^6	1.14×10^6	1.14×10^6
	60	3.5×10^5	5.5×10^5	8×10^5
